# Managing Cancer Pain at the End of Life with Multiple Strong Opioids: A Population-Based Retrospective Cohort Study in Primary Care

**DOI:** 10.1371/journal.pone.0079266

**Published:** 2014-01-27

**Authors:** Wei Gao, Martin Gulliford, Michael I. Bennett, Fliss E. M. Murtagh, Irene J. Higginson

**Affiliations:** 1 King's College London, School of Medicine, Department of Palliative Care, Policy and Rehabilitation, London, United Kingdom; 2 King's College London, School of Medicine, Department of Primary Care and Public Health Sciences, London, United Kingdom; 3 University of Leeds, Leeds Institute of Health Sciences, Academic Unit of Palliative Care, Leeds, United Kingdom; The James Cook University Hospital, United Kingdom

## Abstract

**Background:**

End-of-life cancer patients commonly receive more than one type of strong opioid. The three-step analgesic ladder framework of the World Health Organisation (WHO) provides no guidance on multiple opioid prescribing and there is little epidemiological data available to inform practice. This study aims to investigate the time trend of such cases and the associated factors.

**Methods:**

Strong opioid prescribing in the last three months of life of cancer patients were extracted from the General Practice Research Database (GPRD). The outcome variable was the number of different types of prescribed non-rescue doses of opioids (1 vs 2–4, referred to as a complex case). Associated factors were evaluated using prevalence ratios (PR) derived from multivariate log-binomial model, adjusting for clustering effects and potential confounding variables.

**Results:**

Overall, 26.4% (95% CI: 25.6–27.1%) of 13,427 cancer patients (lung 41.7%, colorectal 19.1%, breast 18.6%, prostate 15.5%, head and neck 5.0%) were complex cases. Complex cases increased steadily over the study period (1.02% annually, 95%CI: 0.42–1.61%, p = 0.048) but with a small dip (7.5% reduction, 95%CI: −0.03 to 17.8%) around the period of the Shipman case, a British primary care doctor who murdered his patients with opioids. The dip significantly affected the correlation of the complex cases with persistent increasing background opioid prescribing (weighted correlation coefficients pre-, post-Shipman periods: 0.98(95%CI: 0.67–1.00), p = 0.011; 0.14 (95%CI: −0.85 to 0.91), p = 0.85). Multivariate adjusted analysis showed that the complex cases were predominantly associated with year of death (PRs vs 2000: 1.05–1.65), not other demographic and clinical factors except colorectal cancer (PR vs lung cancer: 1.24, 95%CI: 1.12–1.37).

**Conclusion:**

These findings suggest that prescribing behaviour, rather than patient factors, plays an important role in multiple opioid prescribing at the end of life; highlighting the need for training and education that goes beyond the well-recognised WHO approach for clinical practitioners.

## Introduction

The reported prevalence of moderate to severe pain in advanced cancer is approximately 64%, with a sharp increase to as high as 80–90% at the end of life [1], [2]. The traditional mainstay of pain management since the 1980s has been the World Health Organization (WHO) three-step “ladder” approach, involving recommendation of a single strong opioid for moderate to severe cancer pain [3], [4]. However, increasingly over the treatment course, patients may be switched (sometimes called rotated) from one opioid to another because of side effects or concerns regarding effectiveness of the initial opioid. In addition, patients may be prescribed combinations of different opioids, usually when they are receiving long acting and short acting compounds or compounds by different routes, especially if one compound is not available across the required formulations or routes. Clinical management in such circumstances is more complex, driven by patient's response, and the need to have both shorter and longer acting preparations and equianalgesic dose ratios. The process of combining or switching opioids is more complex for the clinician—as s/he must understand the different half-life, receptors and conversion ratios of these opioids, which can vary greatly between individuals, opioids and even by opioid dose [5].

A patient being prescribed more than one type of strong opioid (referred to here as a complex case) is a result of interplay between various factors, which may involve disease-, socio-demographic, healthcare practitioners- and health policy-related variables [6]–[12]. Clinical observations found that around 10–30% of cancer patients treated with oral morphine could not reach a balance between sufficient pain control and acceptable level of side effects [13]. These patients need opioid switching or an opioid combination to achieve optimal pain control. However, prescribing more than one type of strong opioid may not always mean that there is a genuine clinical need; it may sometimes reflect inadequate prescribing behaviour of physicians as discussed in a review paper about physician-related barriers to cancer pain management with opioid [14]. For example, in some situations, adequate pain control could have been achieved by escalating the dosage of the existing opioid therapy. The physician may fail to increase doses due to fear of overdose; rather, s/he may choose an alternative opioid at a low dose level. The use of adjuvants can enhance the analgesic effect of opioid drugs in patients with cancer [15]. Concurrent use of adjuvants is recommended by the WHO and has been recognised as one of the effective strategies in improving the balance between analgesia and side effects. However, consistent evidence suggests an underutilisation of adjuvants in cancer pain management, which may contribute to unnecessary opioid switching or rotation [16]. For the reasons discussed above, the term complex case is used here as an indicator of complexity in managing cancer pain at the end of life, although we appreciate that complexity in this context reflects prescribing patterns, which may not be the ‘true’ clinical complexity of the patients.

Studies concerning using opioids for pain relief in cancer predominantly focused on factors affecting whether a patient receives opioids or not. There is a scarcity of research on complex cases in cancer pain management at the population level, more specifically on: 1) time trends of complex cases, and 2) factors associated with a patient being a complex case. The first question has policy relevance, the second is imperative for early identification of patients whose pain management will be genuinely complex, to promote initiation of treatment geared towards best management outcome of cancer pain. There is also no data on whether patient related factors are playing a role in complex cases or if it is predominantly related to healthcare practitioner's characteristics, prompting needs for education, training and even policy-level interventions. Nevertheless, the information is particularly important for advanced cancer patients, to improve quality of life in the last few months of life.

## Data and Methods

### Study design

A population-based, retrospective cohort study in primary care.

### Data source

The study sample was extracted from the UK General Practice Research Database (GPRD), the world's largest primary care research database. It covers a broadly representative 6% of the UK population, and contains individual-level longitudinal data from around 11 million patients registered with over 516 primary care practices throughout the UK. It collects information on demographics, diagnosis, prescribed medications, referrals and almost all activities during GP consultations. The GPRD has set up data quality standards to classify general practices with respect to completeness, continuity, and plausibility of data recording. The practices meet the quality standard are known as the “Up To Standard” (UTS) practices [11], [17].

### Ethics statement

The analysis was based on fully anonymised data; therefore, no ethical approval is required for this study.

### Patient cohort

Inclusion criteria to select patients from the GPRD database were:

1. A diagnosis of one of the most common primary cancers(lung, colorectal, breast, prostate, head and neck), identified with Read/OXMIS codes;
2. Died between 01/01/2000 and 31/12/2008;
3. Registered with an UTS GPRD practices for at least one year;
4. With at least one prescribing history of strong opioids in the last three months of life.

### Strong opioids

We reviewed the following fourteen types of strong opioids which included those listed in the 4.7.2 BNF 6.1 and also those available in the market during the study period: morphine, diamorphine, fentanyl family(alfentanil, fentanyl,remifentanil), oxycodone, buprenorphine, hydromorphone, methadone, others(dextromoramide, didipanone, papaveretum, pentazocine, pethidine, tramadol, tapentadol).

### Variables

The outcome variable was the number of different types of strong opioids a patient received in the last three months of life. This was calculated for each individual patient, by prospectively tracking the prescriptions of strong opioids the patient received in the last three months. “Rescue opioid” prescriptions were excluded, because these were prescribed for prevention purpose and patients may not actually use it [18]. The “rescue opioids” (short-acting or immediate release compound) was identified whenever more than one type of strong opioids was prescribed to a patient on the same date. Out of 68,023 prescriptions containing one of the fourteen types of strong opioids, 6,451(9.5%) were written at the same time, of which 2,701(41.9%) were “rescue opioids”.

The explanatory variables included: age at diagnosis, gender, cancer site, year of death, opioid prescribing history in 3–6 months prior to death, adapted Charlson co-morbidity score [19], social economic status(SES) as measured by quintile of the index of multiple deprivation(IMD, 0 = least deprived to 4 = most deprived), location of region where the practice with which patient was registered. The number of non-opioid prescriptions a patient received in the last three months and the survival time were included as potential confounding variables.

### Statistical analysis

Data was summarised using counts and percentages for categorical variables, mean (SD) and median (range) for continuous data. The 95% confidence interval (CI) of the percentage was calculated using Wilson score method. The simple linear trend in the percentage of patients who received more than one type of strong opioid was explored using weighted piecewise linear regression. We used log-binomial regression models to evaluate which variables were associated with patients having received more than one type of strong opioids. The outcome was modelled as a binary variable (1 versus 2–4). The generalised estimation equation (GEE) was used to account for the clustering effects within practices. The explanatory variables were first tested in the bivariate analysis using Chi-square test for their associations with the outcome variable. Variables that were significant at the cut-off p value of 0.20 were used to build multiple regression models, and the adjusted proportion ratios (APRs) were estimated from the models.

All first-order interaction effects between factors showing significant effect in multiple regression models were investigated.

We performed two sensitivity analyses to test the robustness of the findings of the main analysis: 1) the percentage of the estimated cost by GP prescribed opioids among all opioid prescriptions as an additional adjusted variable, the analysis was restricted to the England regions which have the data [20]; 2) analysis based on patients without any referral records in the last three months - this is in order to eliminate the potential impact by health care from non-GP healthcare professionals or Step III opioid prescribing not captured by the database.

A two-sided p value less than 0.05 was considered statistically significant. Analyses were performed using SAS 9.3(SAS Institute, Cary, North Carolina, USA).

## Results

In total, 13,427 cancer patients with at least one prescribing record of strong opioids in the last three months of life were included in this study. It was a subset from a total of 29,847 patients with a diagnosis of one of the five major cancers, excluding 37 patients with problematic (eg. year of death = 2500 or missing information on key variables (eg. date of diagnosis). The median time to death after the last opioid prescription was 11 days (Mean (SD): 17.9(19.2) days; range: 0–90 days). The median number of non-opioid prescriptions patients received from their GPs in the last three months was 24(Mean (SD): 28.1(21.2); range: 0–446).

Table 1 shows the socio-demographic and clinical characteristics of the study population. Nearly 80% of those with at least one prescribing history of strong opioids were aged over 60. There were slightly more men than women (53.8% versus 46.2%). Two in five (41.7%) patients in the study were lung cancer. The median co-morbidity score was 3.0(Mean (SD): 4.6(2.9): range: 0–16), one in three cancer patients (31.7%) were scored 6 or higher in the co-morbidity index. Two thirds (66.2%) patients had no prescribing history of strong opioids in 3–6 months before death. The study sample consisted more of people who died in recent years (6.7% in 2000 to 15.3% and 12.9% in 2007 and 2008). Slightly more people (SES 5: 24.0% versus 18.1% to 19.8% in other SES strata) were from more deprived areas. Regions contributed differently to the study sample in terms of number of patients (Northern Ireland 2.8% to Southern region 30.5%).

**Table 1:** Demographic and clinical characteristics of cancer patients receiving at least one prescription of strong opioids at the end of life, UK 2000–2008.

Variable	Value	Types of strong opioid prescribed in the last three months			
		1	2	3+	All
	All	9888(73.6)	3113(23.2)	426(3.2)	13427(100.0)
**Age**	<50	779(7.9)	228(7.3)	40(9.4)	1047(7.8)
	50–59	1411(14.3)	455(14.6)	74(17.4)	1940(14.4)
	60–69	2526(25.5)	803(25.8)	120(28.2)	3449(25.7)
	70–79	3121(31.6)	1000(32.1)	130(30.5)	4251(31.7)
	80+	2051(20.7)	627(20.1)	62(14.6)	2740(20.4)
**Gender**	Female	4555(46.1)	1454(46.7)	198(46.5)	6207(46.2)
	Male	5333(53.9)	1659(53.3)	228(53.5)	7220(53.8)
**Cancer site**	Breast	1861(18.8)	554(17.8)	87(20.4)	2502(18.6)
	Colorectal	1827(18.5)	647(20.8)	94(22.1)	2568(19.1)
	Head and neck	490(5.0)	158(5.1)	29(6.8)	677(5.0)
	Lung	4171(42.2)	1279(41.1)	150(35.2)	5600(41.7)
	Prostate	1539(15.6)	475(15.3)	66(15.5)	2080(15.5)
**Co-morbidity score**	0–2	3197(32.3)	986(31.7)	154(36.2)	4337(32.3)
	3–5	3608(36.5)	1097(35.2)	140(32.9)	4845(36.1)
	6–8	1744(17.6)	567(18.2)	71(16.7)	2382(17.7)
	9+	1339(13.5)	463(14.9)	61(14.3)	1863(13.9)
**Prescribing history of opioids 3–6 months before death**	No	6639(67.1)	2001(64.3)	244(57.3)	8884(66.2)
	Yes	3249(32.9)	1112(35.7)	182(42.7)	4543(33.8)
**Year**	2000	726(7.3)	165(5.3)	15(3.5)	1669(12.4)
	2001	860(8.7)	206(6.6)	24(5.6)	3795(28.3)
	2002	908(9.2)	243(7.8)	32(7.5)	3484(25.9)
	2003	979(9.9)	310(10.0)	36(8.5)	2090(15.6)
	2004	1141(11.5)	412(13.2)	63(14.8)	2389(17.8)
	2005	1265(12.8)	413(13.3)	60(14.1)	905(6.7)
	2006	1299(13.1)	422(13.6)	59(13.8)	1090(8.1)
	2007	1489(15.1)	486(15.6)	76(17.8)	1183(8.8)
	2008	1221(12.3)	456(14.6)	61(14.3)	1325(9.9)
**SES**	1 (Least deprived)	1822(18.4)	643(20.7)	78(18.3)	1616(12.0)
	2	1797(18.2)	556(17.9)	75(17.6)	1738(12.9)
	3	1930(19.5)	633(20.3)	91(21.4)	1780(13.3)
	4	1943(19.7)	551(17.7)	80(18.8)	2051(15.3)
	5 (Most deprived)	2396(24.2)	730(23.5)	102(23.9)	1738(12.9)
**Region**	EASTERN	1342(13.6)	424(13.6)	67(15.7)	2543(18.9)
	LONDON	884(8.9)	237(7.6)	21(4.9)	2428(18.1)
	NORTH EAST	815(8.2)	237(7.6)	28(6.6)	2654(19.8)
	NORTH WEST AND WEST MIDLAND	2453(24.8)	790(25.4)	131(30.8)	2574(19.2)
	NORTHERN IRELAND	278(2.8)	91(2.9)	13(3.1)	3228(24.0)
	SCOTLAND	597(6.0)	183(5.9)	25(5.9)	1883(14.0)
	SOUTHERN	2986(30.2)	996(32.0)	115(27.0)	1142(8.5)
	WALES	533(5.4)	155(5.0)	26(6.1)	1080(8.0)

**Figure 1:**
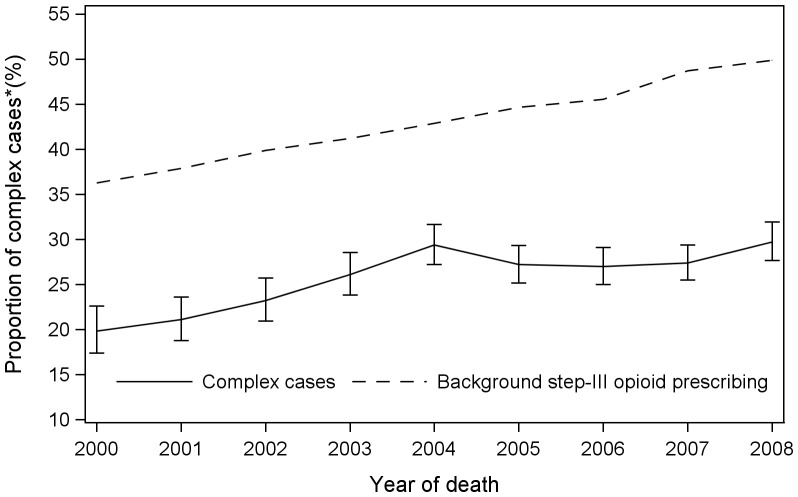
Percentage (95% CI) of patients receiving more than one types of strong opioids in the last three months of life, England 2000–2008 (N = 13, 427). *A complex case refers to a patient being prescribed for more than one type of strong opioids in the last three months of life.

Over the 9-year period, 73.6% (95%CI: 72.9 to 74.4%), 23.2% (95%CI: 22.4 to 23.9%), 3.0%(95%CI: 2.7 to 3.3%) of patients received 1, 2 and 3 different types of strong opioids, respectively; less than 0.2%(95%CI: 0.1 to 0.3%) patients received four types. The proportion of complex cases according to our definition increased steadily over the study period (1.02% annually, 95%CI: 0.42 to 1.61%). The proportion of patients who received more than one type of strong opioid increased from 19.9%(95%CI: 17.4 to 22.6%) in 2000 to 29.4%(95%CI: 27.2 to 31.7%) in 2004(annual increase: 2.1%, 95%CI: 0.7 to 3.5%), followed by small drops in 2005 and 2006 to 27.0%(95%CI: 25.0 to 29.1%); the proportion rose again since 2007, to 29.7%(95%CI: 27.6 to 31.9%) in 2008; the annual increase was 0.2%(95%CI:−0.8 to 1.2%) after 2004. The correlation between background opioid prescribing and the proportion of complex cases was high (weighted correlation coefficient (ρ): 0.82, 95%CI: 0.31 to 0.96, p = 0.004) though differed by period: a nearly perfect correlation before 2004(weighted ρ: 0.98, 95%CI: 0.67 to 1.00, p = 0.011) but non-existent after 2004(weighted ρ: 0.14, 95%CI: −0.85 to 0.91, p = 0.85). A piecewise regression analysis with one breakpoint at 2004 showed a satisfactory fit to the data (F_df = 3_ = 21.5, p = 0.003) (Figure 1). The overall proportion of complex cases varied narrowly by regions, ranging from the lowest 20.2% (95%CI: 20.2–25.1%) in London to the highest 27.3% (95%CI: 25.8–28.8%) in North West and West Midlands.

Among those receiving two types of strong opioids (N = 3,113), three common alternatives to morphine were diamorphine (45.9%, 95%CI: 44.1–47.6%, fentanyl family (20.7%, 95%CI: 19.2–22.1%) and oxycodone (11.5%, 95%CI: 10.4–12.6%). For those who had three types (N = 401), morphine, diamorphine and fentanyl family together accounted for nearly half (45.6%, 95%CI: 40.8 to 50.5%). Only 25 patients were prescribed four types of strong opioids in the last three months of life, 10 of them (40.0%, 95%CI: 20.8 to 59.2%) were prescribed morphine and its three most common alternatives (diamorphine, fentanyl family and oxycodone).

The bivariate association analysis identified 5 out of 8 candidate variables for constructing the multiple regression model (Table 2). The number of non-opioids prescriptions a patient received in the last three months was included as a confounding variable (significant in bivariate and multivariate analysis, both p values<0.001). Only the statistical significance pertaining to cancer site and year of death on the outcome was maintained in multiple regression analysis. Compared with patients with lung cancer, those with colorectal cancer (Adjusted PR: 1.25, 95%CI: 1.13 to 1.39) had higher chance of receiving more than one type of strong opioid. The increasing trend in the chance of patients receiving more than one type of strong opioids was confirmed in the multivariate analysis: the adjusted PR versus reference year (2000) rose to the peak in 2004(1.65, 95%CI: 1.36 to 2.01), declined gradually thereafter, before climbing again to 1.66(95%CI: 1.35 to 2.04) in 2008. The two sensitivity analysis showed similar results to those of main analysis (Table S1, S2). The interaction effect between year of death and cancer site was not significant (P = 0.35, *X* ^2^_df = 32_ = 34.4).

**Table 2:** Crude prevalence ratios (CPR, 95%CI) and adjusted prevalence ratios (APR, 95%CI)* of factors associated with the number of type of strong analgesics a patient received in the last three months of life (N = 13,427).

Characteristics	Value	Crude PR	P value for overall effects	Adjusted PR	P value for Overall effects
**Age**	<50	1.00	0.48	-	-
	50–59	1.10(0.92 to 1.30)		-	-
	60–69	1.06(0.92 to 1.24)		-	-
	70–79	1.05(0.91 to 1.22)		-	-
	80+	0.98(0.83 to 1.15)		-	-
**Gender**	Male	1.00	0.51	-	-
	Female	1.03(0.95 to 1.11)		-	-
**Cancer site**	Lung	1.00	0.022	1.00	<0.001
	Breast	1.00(0.90 to 1.11)		1.05(0.93 to 1.19)	
	Colorectal	1.17(1.06 to 1.30)		1.25(1.13 to 1.39)	
	Head & neck	1.11(0.93 to 1.33)		1.09(0.91 to 1.30)	
	Prostate	1.01(0.90 to 1.14)		0.96(0.85 to 1.10)	
**Co-morbidity score**	0–2	1.00	0.21	-	-
	3–5	0.96(0.88 to 1.04)		-	-
	6–8	1.02(0.92 to 1.14)		-	-
	9–17	1.09(0.96 to 1.23)		-	-
**Prescribing opioids in 3–6 months before death**	No	1.00	<0.001	1.00	0.05
	Yes	1.18(1.08 to 1.29)		1.10((1.00 to 1.21)	
**Year of death**	2000	1.00	<0.001	1.00	<0.001
	2001	1.10(0.90 to 1.35)		1.05(0.86 to 1.29)	
	2002	1.25(1.00 to 1.56)		1.21(0.96 to 1.52)	
	2003	1.45(1.17 to 1.78)		1.42(1.15 to 1.75)	
	2004	1.72(1.42 to 2.09)		1.65(1.36 to 2.01)	
	2005	1.55(1.28 to 1.88)		1.46(1.20 to 1.77)	
	2006	1.54(1.26 to 1.89)		1.49(1.21 to 1.84)	
	2007	1.57(1.29 to 1.90)		1.44(1.18 to 1.75)	
	2008	1.77(1.44 to 2.16)		1.66(1.35 to 2.04)	
**SES**	0 (least deprived)	1.00	0.08	1.00	0.09
	1	0.88(0.76 to 1.03)		0.88(0.75 to 1.02)	
	2	0.93(0.81 to 1.08)		0.93(0.80 to 1.08)	
	3	0.81(0.70 to 0.94)		0.82(0.71 to 0.95)	
	4 (most deprived)	0.87(0.75 to 0.99)		0.86(0.74 to 0.99)	
**Region**	Southern	1.00	0.10	1.00	0.19
	North east	0.88(0.76 to 1.03)		0.94(0.80 to 1.10)	
	Eastern	0.96(0.83 to 1.12)		0.99(0.85 to 1.15)	
	London	0.76(0.64 to 0.91)		0.79(0.66 to 0.95)	
	North west	0.99(0.87 to 1.13)		0.99(0.85 to 1.15)	
	Northern	1.00(0.80 to 1.25)		0.89(0.69 to 1.15)	
	Wales	0.91(0.74 to 1.10)		0.84(0.70 to 1.02)	
	Scotland	0.92(0.75 to 1.15)		0.90(0.72 to 1.14)	

* CPRs and APRs were derived by using log-binomial models with the adjustment of correlation within practices. PRs greater than one indicate that the presence of the characteristic confers higher risk of receiving more types of opioids.

## Discussion

One of the main and interesting findings from this large-scale, population-based longitudinal study is that the “year of death” is the factor most strongly associated with being a complex case, outweighing all other factors. Patients who died in more recent years were more likely to receive more than one type of strong opioids. The number of types of opioids prescribed to the patients remained relatively stable during the study period, ranging from 8 in 1999 to 11 in 2000–2002 and 2006–2008. Over the years, the under-treatment of pain in cancer patients has attracted considerable attention [21]. Most barriers, reported in the literature, to optimal pain management using opioids (ie. misconceptions of patients, reluctance of physicians in prescribing) are concerned with whether a patient receives opioids or not, rather than whether patients have been prescribed more than one type of strong opioid. This study therefore complements existing studies and examines the patients who are already on opioid therapy [2], [18]. However, the barriers identified in the former may still influence dosage, type and combination of opioids; and therefore, affect the chance of a patient being a complex case. A systematic review identified a problem that has not yet been widely researched, regarding the role of the adequacy of opioid prescribing in cancer pain control [14]. Two aspects of evidence arising from this study(as listed below) also suggest that prescribing behaviour plays an important role in patients receiving more types of strong opioids.

Firstly, the proportion of complex cases experienced a small drop at 2005, it was coincident with the Shipman case that came into full light following the suicide of this serial killer GP [22]. The media coverage brought an unprecedented negative image on strong opioids. A study conducted during the same period demonstrated that the Shipman case made health professionals more cautious of using strong opioids for treating pain [23]. Since Dr Harold Shipman killed his patients through administering overdose opioids [22], one would expect that the case would make GPs overcautious in escalating opioid dose or switching to alternative equi-analgesic opioids, even if there was a genuine need of doing so. A recent qualitative study found professionals working in primary care settings had particular concerns about giving high doses opioids and felt incompetent in using opioids [10].

Second, although the increase in complex cases may be associated with the background opioid prescribing, easier access or other factors, it only appeared to be true before the wake of the Shipman case. A dramatic change (from perfect to none) in the correlation between background opioid prescribing and the complex cases was noted around the year of 2005. Patients and their families often have concerns about initiating opioids, which may impact whether a patient does or does not receive opioids. Apparently, Shipman's case did not affect the public's perceptions about opioids, as evident from the steady increase in patients receiving opioid prescriptions [17]. However, after the initiation of opioid therapy, it seems that GPs had more involvement in managing or maintaining the therapy, and there may have been more confusion about which opioid to prescribe in the face of wider availability of different opioids and greater variety of routes of administration.

This identified role of prescribing behaviour in complex cases warrants further in-depth investigation. It may indicate prescribing confusion or inadequacy or some other under-recognised prescribing behaviour. It emphasizes the need for urgent training and education to improve the knowledge and attitude of clinicians about opioids (eg. reduced fear about life-limiting effects when appropriately titrated).

We found that colorectal cancer patients had a higher chance of being a complex case than patients with the other cancers. It may be related to one of the most common side effects resulting from using opioids - constipation. Although it is widely advised that the concurrent use of laxatives and opioids, evidence suggests only a minority of patients who were on strong opioids received laxatives concomitantly [4], [24], [25]. The non-compliance to good prescribing practices poses particular challenges for pain management in cancers with pre-existing constipation, e.g. colorectal cancer. Future work needs to examine how and to what extent suboptimal prescribing leads to a patient having to switch unnecessarily.

Elderly cancer patients are less likely to receive analgesia than their younger counterparts. Earlier research showed older age was independently associated with the under-prescription for all levels of analgesics [11]. Barriers to optimal pain relief may be more common in older than in younger patients, which are mainly related to misconceptions about opioids and disease [7]. However, our data shows that once patients were on opioid therapies, there was no evidence that pain management is more complex or simpler in elderly patients. This is consistent with previous findings [9].

Evidence is now emerging that there is an individual(patient by patient) response to different opioids [26]. Searching for genetic or disease specific modulators of this has so far proven unsuccessful [27]. Current practice recommends switching if patients fail to respond to an opioid, however, there is no evidence that one switch is better than another. Some switches are more complex than others [28], [29]. Switching opioids or combining them requires skilled clinicians who are aware of the latest evidence regarding appropriate doses, as well a carefully monitoring during the switch [26].

Nevertheless, over one in four cancer patients were complex cases, beyond the WHO analgesic ladder. The increasing trend of complex cases and the possible involvement of prescribing practices in the process highlight the urgent need for further investigation to clarify the role of prescribing behaviour and audit opioid prescribing, as well as understand the effectiveness of opioid switching, its mechanisms, and importantly equianalgesic dose ratios. If it was prescribing inadequacy (especially if patients were switched simply because they had not reached an effective dose without side effects in the original opioid), then this prompts a need for education and training [12], [30], [31]. The educational or interventional programmes should target not only GPs, but also a range of health professionals, who may play an even more important role in initiating and maintaining opioid therapies [10], [31]. The education and training should go beyond the well-recognised WHO approach, and focus on issues of equi-analgesics and safety, which are most challenging when converting from one opioid to another or using opioids in combination.

Several limitations should be noted to interpret our findings. First, the GPRD database only captures the opioids prescribed by the GPs, we may miss some prescriptions through non-GP routes. The likely impact of these “missed opioids” would be underestimating the problem of the complex cases, which will strengthen the need for educating and training health professionals. The sensitivity analysis including the percentage of the GP prescribing in the multiple regression model did not substantially change the main findings. Second, there is no data available to assess the needs of pain relief and the effectiveness of the pain medications, which are important for effective pain management. Though it is reasonable to assume that those who were receiving opioid prescriptions should be the group with high needs, we won't be able to judge the relationship between “more types of opioids” and the outcome of pain treatment.

In conclusion, this large scale, population-based retrospective cohort study found over one in four cancer patients were complex cases; this showed an overall increasing trend but with a small dip around the period of the Shipman case, a British primary care doctor who murdered his patients with opioids. The dip significantly affected the correlation of complex case with persistent increasing background opioid prescribing. Multivariate adjusted analysis also showed that complex cases were predominantly associated with year of death, not other demographic and clinical factors except colorectal cancer. These findings suggest that prescribing behaviour, rather than patient factors, plays an important role in multiple opioid prescribing at the end of life; highlighting the need for training and education that goes beyond the well-recognised WHO approach for clinical practitioners.

## Supporting Information

Table S1 Crude prevalence ratios (CPR, 95%CI) and adjusted prevalence ratios (APR, 95%CI)* of factors associated with the number of type of strong analgesics a patient received in the last three months of life (N = 11,526), adjusting for level of opioid costs accounted for in each English region**.
(DOCX)

Table S2 Crude prevalence ratios (CPR, 95%CI) and adjusted prevalence ratios (APR, 95%CI)* of factors associated with the number of type of strong opioids a patient received in the last three months of life (N = 8,712), based on patients with no referral record during the last three months.
(DOCX)
