# Supplementary material for: Managing Cancer Pain at the End of Life with Multiple Strong Opioids: A Population-Based Retrospective Cohort Study in Primary Care
Source: PLoS One. 2014 Jan 27;9(1):e79266. doi: 10.1371/journal.pone.0079266 (PMC3903468; doi:10.1371/journal.pone.0079266)
Supplement: Table S1 — Crude prevalence ratios (CPR, 95%CI) and adjusted prevalence ratios (APR, 95%CI)* of factors associated with the number of type of strong analgesics a patient received in the last three months of life (N = 11,526), adjusting for level of opioid costs accounted for in each English region**. (DOCX) [file pone.0079266.s001.docx]

Table S1 Crude prevalence ratios (CPR, 95%CI) and adjusted prevalence ratios (APR, 95%CI)* of factors associated with the number of type of strong analgesics a patient received in the last three months of life (N=11,526), adjusting for level of opioid costs accounted for in each English region**

| **Characteristics** | **Value** | **Crude PR** | **P value for**  **overall effects** | **Adjusted PR** | **P value for**  **Overall effects** |
| --- | --- | --- | --- | --- | --- |
| Age | <50 | 1.00 | 0.81 | - | - |
|  | 50-59 | 1.04(0.87 to 1.25) |  | - | - |
|  | 60-69 | 1.05(0.89 to 1.22) |  | - | - |
|  | 70-79 | 1.01(0.87 to 1.18) |  | - | - |
|  | 80+ | 0.97(0.81 to 1.15) |  | - | - |
| Gender | Male | 1.00 | 0.30 | - | - |
|  | Female | 1.04(0.96 to 1.13) |  | - | - |
| Cancer site | Lung | 1.00 | 0.24 | 1.00 | 0.005 |
|  | Breast | 1.02(0.91 to 1.15) |  | 1.09(0.96 to 1.24) |  |
|  | Colorectal | 1.11(1.01 to 1.24) |  | 1.19(1.07 to 1.33) |  |
|  | Head & neck | 1.08(0.89 to 1.31) |  | 1.07(0.88 to 1.31) |  |
|  | Prostate | 0.98(0.86 to 1.11) |  | 0.94(0.82 to 1.07) |  |
| Co-morbidity score | 0-2 | 1.00 | 0.57 | - | - |
|  | 3-5 | 0.97(0.88 to 1.07) |  | - | - |
|  | 6-8 | 1.05(0.93 to 1.17) |  | - | - |
|  | 9-17 | 1.05(0.91 to 1.20) |  | - | - |
| Prescribing opioids in  3-6 months before death | No | 1.00 | <0.001 | 1.00 | 0.05 |
|  | Yes | 1.19(1.08 to 1.32) |  | 1.11(1.00 to 1.23) |  |
| Year of death | 2000 | 1.00 | <0.001 | 1.00 | <0.001 |
|  | 2001 | 1.07(0.86 to 1.32) |  | 1.02(0.82 to 1.26) |  |
|  | 2002 | 1.24(0.98 to 1.58) |  | 1.20(0.94 to 1.54) |  |
|  | 2003 | 1.40(1.12 to 1.74) |  | 1.37(1.10 to 1.71) |  |
|  | 2004 | 1.68(1.37 to 2.08) |  | 1.63(1.32 to 2.01) |  |
|  | 2005 | 1.52(1.24 to 1.86) |  | 1.43(1.17 to 1.76) |  |
|  | 2006 | 1.58(1.28 to 1.96) |  | 1.53(1.23 to 1.90) |  |
|  | 2007 | 1.57(1.28 to 1.92) |  | 1.45(1.18 to 1.78) |  |
|  | 2008 | 1.72(1.39 to 2.13) |  | 1.60(1.29 to 1.99) |  |
| SES | 0 (least deprived) | 1.00 | 0.035 | 1.00 | 0.036 |
|  | 1 | 0.84(0.71 to 0.99) |  | 0.84(0.71 to 0.99) |  |
|  | 2 | 0.92(0.79 to 1.08) |  | 0.93(0.79 to 1.09) |  |
|  | 3 | 0.79(0.67 to 0.93) |  | 0.80(0.68 to 0.94) |  |
|  | 4 (most deprived) | 0.82(0.70 to 0.96) |  | 0.80(0.68 to 0.95) |  |
| Region | Southern | 1.00 | 0.022 | 1.00 | 0.79 |
|  | North east | 0.88(0.76 to 1.03) |  | 1.01(0.88 to 1.16) |  |
|  | Eastern | 0.96(0.83 to 1.12) |  | 0.95(0.81 to 1.12) |  |
|  | London | 0.76(0.64 to 0.91) |  | 0.80(0.67 to 0.95) |  |
|  | North west | 0.99(0.87 to 1.13) |  | 0.99(0.85 to 1.15) |  |

*CPRs and APRs were derived by using log-binomial models with the adjustment of correlation within practices. PRs greater than one indicate that the presence of the characteristic confers higher risk of receiving more types of opioids. Multiple regression model was adjusted for an additional variable--Percentage of costs of total opioid prescribing by primary care. Varies by region with North East 83.7% to South West 76.6%, London is lower at 62.3% (Hospital Prescribing, 2005), which may account for the apparent lower prescribing of opioids in London. However, London also has a greater drug abuse population, which are prescribed methadone from hospital clinics. ** Analyses were only restricted to regions with prescribing cost data.
