# Supplementary material for: Managing Cancer Pain at the End of Life with Multiple Strong Opioids: A Population-Based Retrospective Cohort Study in Primary Care
Source: PLoS One. 2014 Jan 27;9(1):e79266. doi: 10.1371/journal.pone.0079266 (PMC3903468; doi:10.1371/journal.pone.0079266)
Supplement: Table S2 — Crude prevalence ratios (CPR, 95%CI) and adjusted prevalence ratios (APR, 95%CI)* of factors associated with the number of type of strong opioids a patient received in the last three months of life (N = 8,712), based on patients with no referral record during the last three months. (DOCX) [file pone.0079266.s002.docx]

Table S2 Crude prevalence ratios (CPR, 95%CI) and adjusted prevalence ratios (APR, 95%CI)* of factors associated with the number of type of strong opioids a patient received in the last three months of life (N=8,712), based on patients with no referral record during the last three months

| **Characteristics** | **Value** | **Crude PR** | **P value for**  **overall effects** | **Adjusted PR** | **P value for**  **Overall effects** |
| --- | --- | --- | --- | --- | --- |
| Age | <50 | 1.00 | 0.51 | - | - |
|  | 50-59 | 1.06(0.86 to 1.31) |  | - | - |
|  | 60-69 | 1.09(0.91 to 1.30) |  | - | - |
|  | 70-79 | 1.10(0.92 to 1.32) |  | - | - |
|  | 80+ | 0.99(0.81 to 1.21) |  | - | - |
| Gender | Male | 1.00 | 0.63 | - | - |
|  | Female | 1.02(0.93 to 1.12) |  | - | - |
| Cancer site | Lung | 1.00 | 0.34 | 1.00 | 0.035 |
|  | Breast | 1.03(0.90 to 1.17) |  | 1.09(0.94 to 1.27) |  |
|  | Colorectal | 1.14(1.00 to 1.28) |  | 1.20(1.06 to 1.37) |  |
|  | Head & neck | 1.08(0.86 to 1.36) |  | 1.05(0.83 to 1.32) |  |
|  | Prostate | 0.99(0.85 to 1.16) |  | 0.95(0.81 to 1.12) |  |
| Co-morbidity score | 0-2 | 1.00 | 0.55 | - | - |
|  | 3-5 | 0.96(0.85 to 1.08) |  | - | - |
|  | 6-8 | 1.04(0.91 to 1.19) |  | - | - |
|  | 9-17 | 1.05(0.88 to 1.24) |  | - | - |
| Prescribing opioids in  3-6 months before death | No | 1.00 | 0.013 | 1.00 | 0.36 |
|  | Yes | 1.14(1.03 to 1.26) |  | 1.05(0.94 to 1.17) |  |
| Year of death | 2000 | 1.00 | <0.001 | 1.00 | <0.001 |
|  | 2001 | 1.07(0.83 to 1.39) |  | 1.02(0.79 to 1.33) |  |
|  | 2002 | 1.32(1.02 to 1.71) |  | 1.26(0.97 to 1.65) |  |
|  | 2003 | 1.50(1.15 to 1.94) |  | 1.45(1.11 to 1.89) |  |
|  | 2004 | 1.86(1.46 to 2.36) |  | 1.76(1.38 to 2.25) |  |
|  | 2005 | 1.71(1.36 to 2.17) |  | 1.61(1.27 to 2.04) |  |
|  | 2006 | 1.64(1.29 to 2.10) |  | 1.58(1.23 to 2.03) |  |
|  | 2007 | 1.66(1.31 to 2.10) |  | 1.48(1.16 to 1.89) |  |
|  | 2008 | 1.92(1.50 to 2.46) |  | 1.78(1.38 to 2.30) |  |
| SES | 0 (least deprived) | 1.00 | 0.16 | 1.00 | 0.17 |
|  | 1 | 0.87(0.73 to 1.05) |  | 0.88(0.73 to 1.05) |  |
|  | 2 | 0.96(0.81 to 1.15) |  | 0.97(0.81 to 1.15) |  |
|  | 3 | 0.85(0.70 to 1.02) |  | 0.85(0.71 to 1.02) |  |
|  | 4 (most deprived) | 0.83(0.70 to 0.98) |  | 0.82(0.69 to 0.98) |  |
| Region | Southern | 1.00 | 0.048 | 1.00 | 0.18 |
|  | North east | 0.79(0.65 to 0.97) |  | 0.85(0.69 to 1.05) |  |
|  | Eastern | 0.91(0.77 to 1.08) |  | 0.92(0.77 to 1.10) |  |
|  | London | 0.70(0.56 to 0.87) |  | 0.72(0.57 to 0.90) |  |
|  | North west | 0.93(0.79 to 1.10) |  | 0.94(0.79 to 1.12) |  |
|  | Northern | 0.91(0.67 to 1.22) |  | 0.80(0.58 to 1.10) |  |
|  | Wales | 0.99(0.80 to 1.22) |  | 0.90(0.72 to 1.13) |  |
|  | Scotland | 0.90(0.69 to 1.17) |  | 0.89(0.67 to 1.19) |  |

*CPRs and APRs were derived by using log-binomial models with the adjustment of correlation within practices. PRs greater than one indicate that the presence of the characteristic confers higher risk of receiving more types of opioids. Analysis was based on patients with no referral record in the last three months.
